# Extracellular vesicles derived from mesenchymal stem cells — a novel therapeutic tool in infectious diseases

**DOI:** 10.1186/s41232-023-00266-6

**Published:** 2023-02-28

**Authors:** Tasaduq Manzoor, Afnan Saleem, Nida Farooq, Lateef Ahmad Dar, Junaid Nazir, Sahar Saleem, Sameena Ismail, Mudasir Bashir Gugjoo, Parvaiz A. Shiekh, Syed Mudasir Ahmad

**Affiliations:** 1 Division of Animal Biotechnology, Faculty of Veterinary Sciences & Animal Husbandry, SKUAST-Kashmir, Srinagar, 190006 India; 2 Government Degree College, Khanabal Kashmir, India; 3 Veterinary Clinical Services Complex, Faculty of Veterinary Sciences & Animal Husbandry, SKUAST-Kashmir, Srinagar, India; 4 Centre for Biomedical Engineering, Indian Institute of Technology-Delhi, New Delhi, 110016 India

**Keywords:** Mesenchymal stem cells, Extracellular vesicles, Wound infections, Exosomes, Stem cell therapy

## Abstract

Extracellular vesicles (EVs) are nano-sized lipid-bilayer encapsulated vesicles produced by the cells. These EVs are released into the surrounding space by almost all cell types. The EVs help in intercellular communication via their payloads which contain various proteins, lipids, and nucleic acids generated from the donor cells and allow for synergistic responses in surrounding cells. In recent years, EVs have been increasingly important in treating infectious diseases, including respiratory tract infections, urinary tract infections, wound infections, sepsis, and intestinal infections. Studies have confirmed the therapeutic value of mesenchymal stem cell-derived EVs (MSC-EVs) for treating infectious diseases to eliminate the pathogen, modulate the resistance, and restore tissue damage in infectious diseases. This can be achieved by producing antimicrobial substances, inhibiting pathogen multiplication, and activating macrophage phagocytic activity. Pathogen compounds can be diffused by inserting them into EVs produced and secreted by host cells or by secreting them as microbial cells producing EVs carrying signalling molecules and DNA shielding infected pathogens from immune attack. EVs play a key role in infectious pathogenesis and hold great promise for developing innovative treatments. In this review, we discuss the role of MSC-EVs in treating various infectious diseases.

## Background

Infectious diseases can cause a variety of health outcomes, ranging from mild illness to significant morbidity and mortality. Infectious diseases are a longstanding and severe public health issue that affects people all over the world. New infectious diseases are arising, and existing ones that were supposed to be under control are restoring strength. The failure of commonly used therapeutic approaches and an increase in the number of severe infectious disease outbreaks have increased the need for alternative therapeutic ways to address infections. Furthermore, it is essential to secure worldwide public health security as the world is approaching an era of extensive antibiotic resistance and the increasing COVID-19 epidemic [1]. Anti-infectious drug development represents a significant advance in the fight against infectious diseases. However, the efficiency of existing anti-infective drugs is fading, as anti-infective agents constantly exert selective pressure on mutations in drug target genes [2]. The effectiveness of current antibiotic treatment is gravely threatened by antibiotic resistance. Developing a unique therapy that does not make resistance worse is crucial. EVs from mesenchymal stem cells (MSCs) are used as an exploratory and promising tool for treating infectious diseases.

EVs are produced by almost all cell types, such as mesenchymal stromal cells, endothelial cells, neurons, B and T cells, dendritic cells, platelets, Schwann cells, and intestinal epithelial cells [3, 4]. These are also found in body fluids like milk, saliva, urine, synovial, and cerebrospinal fluids [5]. Furthermore, the administration route for EVs is quite flexible. EVs can be supplied as intravenous injections. Other delivery methods include direct tissue injections, intraperitoneal injections, and subcutaneous interventions (most of which have been used in studies on tissue repair). Furthermore, EVs are also implanted on scaffolds, and the intratracheal route of administration has been utilised for effective lung diffusion [6].

MSCs are the multipotent population of stromal cells having a wide range of biological functions like multilineage differentiation, anti-inflammatory, immunosuppression, and neuroprotection [7]. MSCs play a vital role in tissue regeneration and homeostasis, making them a promising therapeutic alternative for various diseases [8]. Recent research suggests MSC’s therapeutic effects are due to the released substances. MSCs have a strong paracrine function which is also the key to their therapeutic efficacy. The paracrine effect of MSCs is induced by the release of cytokines, growth factors, and exosomes [9, 10]. The importance of EVs comes from their ability to convey information to target cells, altering the behaviour of the recipient cell transporting protein and nucleic acid payloads such as messenger RNA (mRNA) and micro RNA (miRNA) from one cell to another in a highly selective manner [11]. These EVs are thought to be stable and capable of modulating cellular response in target cells. To enter target cells, EVs use particular receptors. Once recipient cells take up EVs, their biomolecules can control various activities, including gene expression, crucial enzymatic activities, signalling cascades and other mechanisms [12]. MSC-EVs are liable to become an innovative and more effective cell-free therapeutic approach for infectious diseases.

### Classification of extracellular vesicles

Exosomes, microvesicles, and apoptotic bodies are the three EV subtypes characterised based on their size and biogenesis [13, 14] (Table 1). In all three EV subtypes, a lipid bilayer membrane covers a specific payload of biomolecules, such as proteins, RNA, or cellular waste. Exosomes are EVs with a 30–150 nm diameter generated from multivesicular endosome pathways [4, 28]. Exocytosis of multivesicular bodies (MVBs), the key intermediates in endolysosomal transport, releases exosomes both constitutively and upon stimulation [29]. Ceramide is essential for exosome secretion through the local synthesis of its metabolite sphingosine-1-phosphate (S1P) [30]. Several endosomal sorting complexes required for transport (ESCRT) proteins like Alix, Hrs, and TSG101 have been implicated in lysosome function and exosome release [31–33]. Exosomes can transport mRNA, miRNA, oncogenic receptors, and HIV particles horizontally [34–36].

**Table 1:** Classification of EVs

Conventional classification				As per ISEV guidelines
Parameters	Exosomes	Microvesicles	Apoptotic bodies	
Size	30–150 nm	0.1–1 μm	1–5 μm	Small EVs/sEVs (< 200 nm diameter) Large EVs/lEVs (> 200 nm diameter)
Density	1.13–1.19 g/ml	Not well defined	1.16–1.28 g/ml	Low-density EVs (1.1 to 1.2 g/mL) Medium-density EVs (1.16 g/mL) High-density EVs (1.24–1.28 g/mL)
Morphology	Cup shaped	Heterogeneous	Heterogeneous	
Origin	Endosomal	Plasma Membrane	Apoptotic cell	
Composition	Lipids, proteins, miRNA, mRNA	Lipids, proteins, miRNA, mRNA	Lipids, proteins, DNA, miRNA, mRNA	CD63+ stained EVs CD81+ stained EVs Annexin V stained EVs
Lipids	Cholesterol, ceramide, low phosphatidyl serine exposure, Lyobisphosphatidic acid, Sphingomyelin,	Cholesterol, high phosphatidyl serine exposure	High phosphatidyl serine exposure	
Proteins	CD9, CD63, CD81, Annexins, HSPs, Alix, TSG 101, Clathrin, Caveolin, Integrins	CD40, selectins, integrins, flotillins, metalloproteinases	Histones, C3b, thrombospondins	
Cells of origin/biogenesis conditions				Hypoxic EVs Podocyte EVs Apoptotic bodies Large oncosomes
References	[15–19]	[16, 18, 20, 21]	[10, 22, 23]	[24–27]

Microvesicles (MVs) are phospholipid bilayer-encased structures having 100–1000 nm diameter with their size overlapping that of bacteria [13, 37]. The plasma membrane’s controlled release generates them via budding or lipid rafts [38]. The molecular makeup of MVs is still completely unknown, but matrix metalloproteinases (MMPs), glycoproteins, and integrins appear to be abundant in MVs depending on the cell type [39, 40]. MVs, also known as oncosomes, are released by cancer cells. Large oncosomes can be observed in size from 1 to 10 μm [41]. Oncosomes may stimulate organotropic metastatic spread by modulating their target cells’ metabolic and genetic capacities, conferring proteolytic activity and stimulating incursion [42–44]. Apoptotic bodies are the most heterogeneous category of EVs with a wide range of morphology and are generated during apoptosis [45, 46]. They have a diameter of 1–5 μm in the range of platelet size [47]. Apoptosis assures that aged, injured, infected or abnormal cells are selectively removed from normal tissue. Apoptosis is the coordinated disintegration of a cell with cellular debris packed into apoptotic bodies.

The use of the terms described above for the classification of EVs is discouraged due to the significant redundancy among different categories of EVs and the insufficient consensus on precise surface markers. As a result, the International Society for Extracellular Vesicles (ISEV) released the current recommendations in 2018. ISEV recommends the term “extracellular vesicles” as a broad term for nano-sized particles that are normally produced by the cells and are bordered by a lipid bilayer. The current guidelines of ISEV classify the EVs on the basis of (i) size into small EVs (sEVs: diameter of < 200 nm) and medium/large EVs (m/lEVs: diameter > 200 nm) or density with low-density EVs (1.1 to 1.2 g/mL), medium-density EVs (1.16 g/mL), and high-density EVs (1.24–1.28 g/mL); (ii) biological makeup (CD63+ or CD81+ stained EVs, Annexin V stained EVs);and (iii) exosome and microvesicles descriptions should be replaced with explanations of specific biogenesis conditions or cells of origin such as hypoxic EVs, podocyte EVs, apoptotic bodies and large oncosomes [24–27].

Differential ultracentrifugation, filtration, density gradients and immunoaffinity-based isolation procedures are some of the known isolation techniques for EVs [15]. “Ultracentrifugation-linked immunoprecipitation method” is currently the best approach for EV isolation [16, 17]. Furthermore, the different ways for their identification include transmission electron microscope (TEM), nanoparticle tracking analysis (NTA), Western blotting, and recently developed atomic force microscope-infrared spectroscopy (AFM-IR) [18, 19, 24].

The traditional technique for EV isolation makes use of centrifugation to separate particles based on their buoyant density. Following ultracentrifugation, the prepared EV is filtered, and the separated microparticles are chosen based on their size using microfiltration employing filters with different pore sizes. Notably, washing and microfiltration are further EV purification steps that not only improve the EVs’ purity but also reduce their amount. The procedures that combine microfiltration with ultracentrifugation successfully and selectively separate different fractions of microvesicles and exosomes. However, an effective, quick, nearly loss-free and very reproducible approach that enables the isolation of EVs is gel filtration chromatography. The limited yield and relatively high cost of the chromatographic sorbents used in gel chromatography are its drawbacks. The acquired exosomal fraction is also diluted and may need to be concentrated for some applications that follow later. PEG precipitation allows for the simultaneous processing of several samples. This method is most appealing for clinical research because it is easy, quick and affordable, does not distort EVs and needs no extra equipment for isolation, but the sample may be contaminated by various substances like other proteins on a regular basis. To address the issue of protein contamination in the EV fraction, a novel technique for EV isolation utilising a two-phase system with PEG and dextran is provided. In comparison with ultracentrifugation and other kits available, the PEG-dextran solution greatly increases the efficiency of EV isolation, giving the EVs a size and morphology that is similar to that achieved by ultracentrifugation and maintaining the integrity of lipid membranes [20]. There is yet another technique that can be accomplished using affinity-based EV capture using Abs that are specific for a part of the exosome cargo. The clearest illustration of this strategy was provided by Melo and colleagues, who isolated pancreatic cancer-derived exosomes from plasma of a patient and investigated them as prognostic biomarkers of the course and outcome of the disease using immunological capture using Abs-specific for glypican-1 [21]. A better way for isolating EVs should be easy to use, affordable and not require specialised or expensive equipment. It should also be quick and enable isolating EVs from a large sample size. A top objective right now is to develop such EV isolation techniques in order to use them in various clinical and experimental studies.

### Biogenesis of extracellular vesicles

Multiple processes may control the biogenesis and release of EVs from the cell. MVs are formed in areas where there is a significant membrane blebbing [22, 23]. Phospholipase D is activated by ADP-ribosylation factor 6 (ARF6), which is accompanied by an extracellular signal-regulated kinase (ERK). The ERK is trafficked to the plasma membrane, subsequently phosphorylating and activating myosin light chain kinase (MLCK). Finally, MLCK phosphorylates the myosin light chain, which causes the MVs to be released [48]. The ESCRT produces MVs and MVBs. ESCRT is divided into four categories (ESCRT 0, I, II, III) and comprises other proteins like Alix, VPS4, and TSG101. The endosomal membrane is being searched for ubiquitinated transmembrane proteins by the ESCRT 0 complex. The membrane is deformed by ESCRT I and ESCRT II to generate buds with sorted payloads. The ESCRT III causes vesicle scission [49, 50]. The blebbing of the plasma membrane is influenced by various factors that affect membrane deformability and folding, including the lipid content and peripheral cytoskeleton organisation, which modify membrane deformability. MVs have been reported to be abundant in the lipids like phosphatidylserine, lysophosphatidylcholines, sphingomyelins and acylcarnitines [48, 51]. Cholesterol, a significant plasma membrane lipid, is expected to play a key role in MVs formation, and the formation of MVs is reduced when it is depleted [52]. Ceramide that facilitates membrane bending has also been found to control the generation of MVs [53]. The bleb must be squeezed-free from the cell once it has been loaded with payloads by a process mediated by acto-myosin contraction. The downstream kinases like Rho-associated coiled-coil containing kinases (ROCK) and extracellular signal-regulated kinases (ERK) have been shown to increase MV formation when RhoA activity is high [54].

**Fig. 1:**
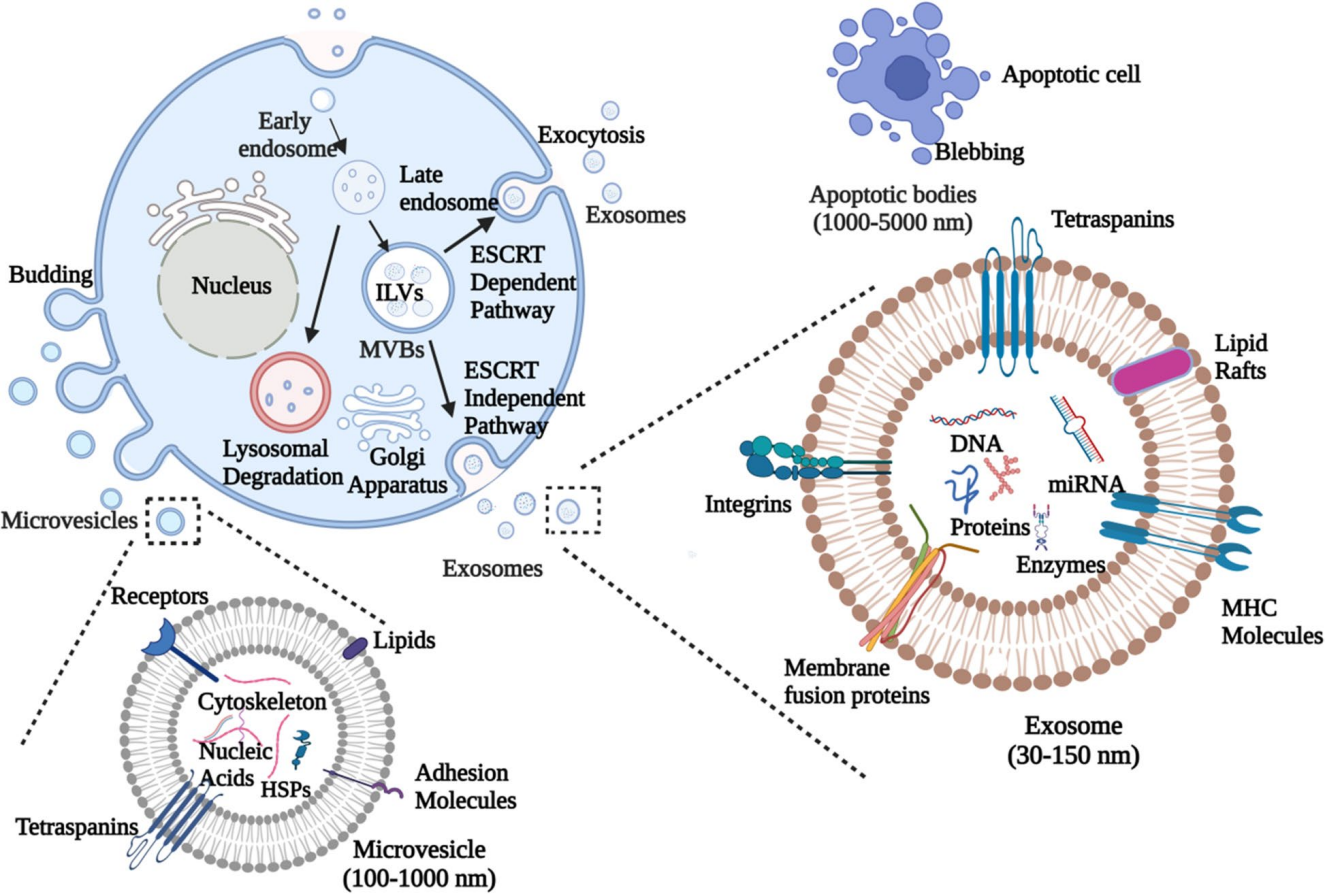
Biogenesis of extracellular vesicles. Exosomes are generated by budding of early endosomes into intraluminal vesicles (ILVs) that are released upon fusion of multivesicular bodies (MVBs) with plasma membrane. MVBs usually have one of two destinies either lysosomal degradation or their release on fusion with plasma membrane, allowing their contents to be released into the extracellular environment. This release can be either ESCRT dependent or ESCRT independent. Microvesicles are generated by outward budding of the plasma membrane into the extracellular region. Apoptotic bodies are formed by the blebbing of plasma membrane from apoptotic cells

Exosomes are formed by the inward budding of the plasma membrane to produce early endosomes [55]. The early endosomal membrane partially invaginates and buds into adjacent lumina with intracellular content to form intraluminal vesicles (ILVs) [56, 57]. Reports suggest that many ILVs are found in late endosomal structures, known as multivesicular bodies (MVBs). These MVBs can adhere to lysosomes for breakdown or bind to the plasma membrane, which releases exosomes into the extracellular space [58] (Fig. 1). MVBs which are rich in cholesterol are assigned to secretion. In contrast, those who are poor in cholesterol are assigned to lysosome breakdown [59]. The formation of ESCRT is required for exosome formation and secretion [60]. The syndecan-syntenin-ALIX pathway allows ESCRT to generate MVBs. Exosome production is aided by syndecan heparan sulphate proteoglycans which interact with syntenin-1 and the ESCRT’s accessory component Alix [61]. Alix, a characteristic exosomal protein linked to multiple ESCRT proteins (TSG101 and CHMP4), has been reported to play a role in the budding and abscission of the endosomal membrane and the selection of exosomal content via interaction with syndecan [62]. There are, however, ESCRT independent mechanisms for the formation of ILVs, such as tetraspanin complex oligomerisation, ceramide production catalysing sphingomyelinase pathway or ILV budding mediated by phospholipase D2 and ARF6 [63–66]. Different mechanisms for producing MVBs and ILVs depend on the cargo sorted within the respective vesicles [67].

Apoptotic bodies are the products of apoptosis-programmed cell death. Several changes occur in an apoptotic cell, starting with nuclear chromatin condensation followed by membrane blebbing and protrusion formation and finally breakdown of the cellular content into discrete membrane-contained vesicles known as apoptotic bodies [68]. Macrophages phagocytise and locally remove most apoptotic bodies during normal development [69]. This removal is mediated by the interactions between phagocyte recognition receptors and specific changes in the constitution of the membrane of apoptotic cells [70]. During the production of apoptotic bodies, phosphatidylserines are translocated to the outer leaflet of the lipid bilayer. Annexin V, T-cell immunoglobulin mucin 4 (TIM4), or milk fat globule-EGF factor 8, recognised by phagocytes, bind to these translocated phosphatidylserines [71, 72]. The oxidation of surface molecules is another well-studied membrane change which helps create binding sites for thrombospondin (TSP) or the complement protein C3b. The receptors, in turn, recognise TSP and C3b on phagocytes [73].

### MSC-EVs in infectious diseases

**Fig. 2:**
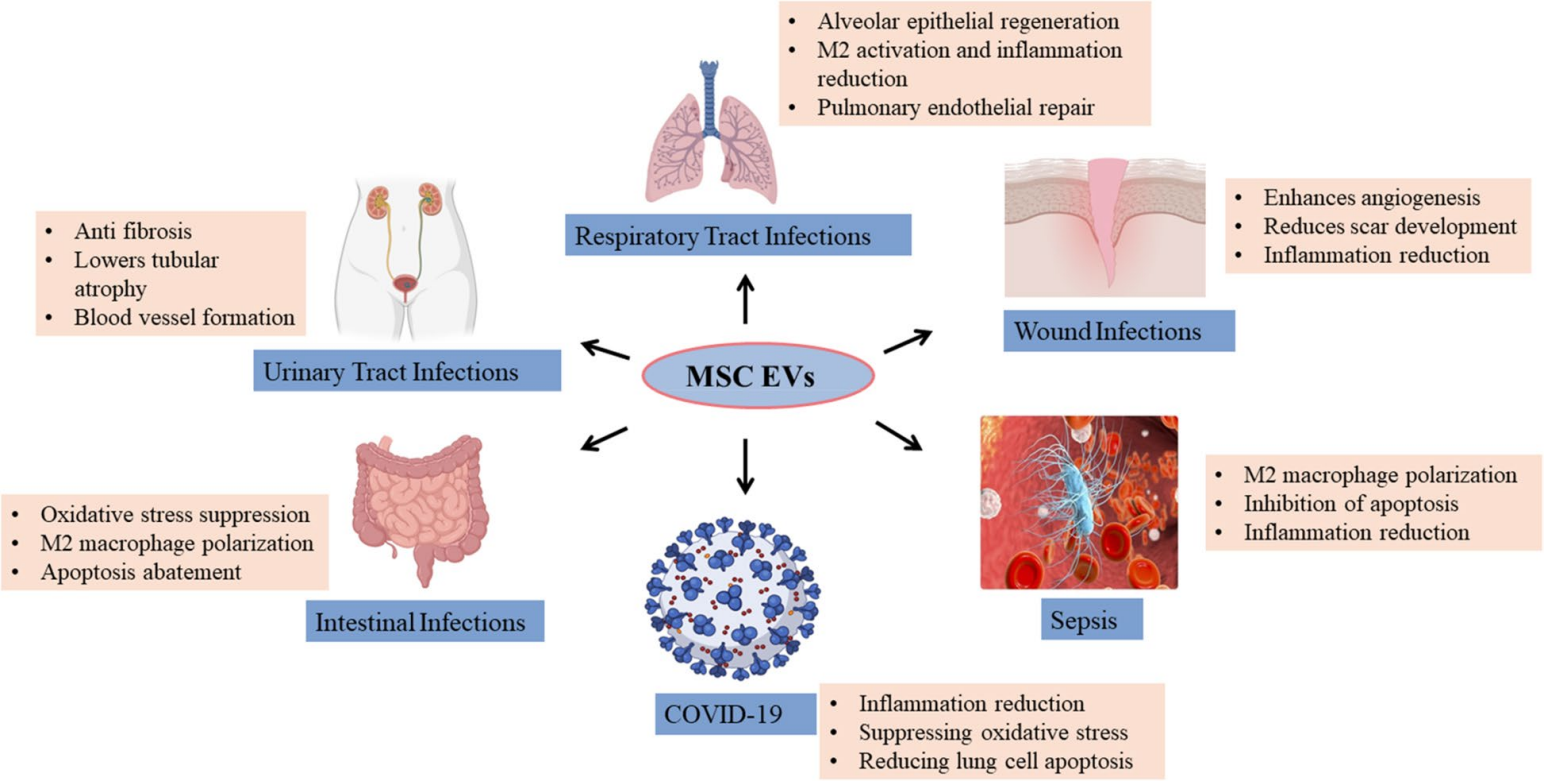
Potential therapeutic role of MSC-EVs in infectious diseases

MSC-EVs have several advantages over MSCs in terms of efficacy and safety that they are efficiently circulating and can penetrate biological barriers such as the blood-brain barrier, reduced carcinogenesis, and stable characteristics [74, 75]. MSC have been shown to have regenerative as well as immunomodulatory effect. However, immune rejection can occur in engrafted MSCs, or the target tissue milieu may be unsuitable for them to operate normally. On the other hand, EVs cannot grow or differentiate inside the human body, posing fewer safety problems and ethical and legal issues. The lack of MSC-EVs’ ability to self-replicate lowers the possibility of tumours as well. Furthermore, EVs can maintain high activity levels at low temperatures [76, 77]. MSC-EVs exhibit antibacterial, immunomodulatory, antiapoptotic and antifibrotic properties and can heal injured tissue [78–80]. In comparison with MSCs, MSC-EVs maintain MSC-like biological functions while being more stable and less prone to tumorigenesis, making them an attractive choice for treating different infections [81]. EVs derived from MSCs are used in gene and cell-based treatments as a tailored delivery mechanism for foreign biological and chemical substances [79, 82]. EVs from MSCs are gaining popularity as they minimise several risks associated with MSC-based treatment (Fig. 2).

### Respiratory tract infection

The role of EVs in lung diseases has previously been investigated using EVs extracted from bronchial-alveolar lavage fluid (BALF) and lung-derived cells [83, 84]. In several respiratory disorders, EVs have been discovered to play a role. Acute lung injury (ALI) and acute respiratory distress syndrome (ARDS) have been the most thoroughly researched diseases [85]. (ARDS)/(ALI) is a heterogeneous condition marked by extensive endothelial and epithelial damage and a robust inflammatory response [86]. MSC-based therapy has shown promise in pre-clinical models of ALI/ARDS due to its immunomodulation and tissue healing abilities [87]. Since MSCs have low differentiation and engraftment efficacy and a significant tumorigenicity risk, researchers have proposed MSC-EVs as a new cell-free treatment for ALI/ARDS [88]. The significant benefits of MSC-EVs on ALI/ARDS include inflammation reduction, alveolar epithelial regeneration, and improved pulmonary endothelial repair [89].

In a study on bronchopulmonary dysplasia (BPD), including hyperoxia exposure in rat models, intratracheally (IT) administered EVs secreted by MSCs were shown to have an early therapeutic impact. These findings support that IT-administered EVs could prevent/treat BPD by improving defective alveolarisation and pulmonary artery remodelling over time. Anti-inflammatory and proliferative processes may be involved in M2 macrophage polarisation [90, 91]. EVs, specifically MVs, are expected to assist in the recruitment of M1 macrophages to injured epithelial cells, suggesting their role in the beginning and maintaining systemic inflammation in the lung epithelium in case of acute lung injury/inflammation [92, 93]. Intratracheal instillation of MSC-derived EVs decreased extracellular pulmonary water, decreased pulmonary congestion, and reduced pulmonary protein porosity in *E. coli* endotoxin-induced lung damage [94]. In addition, these MVs lowered macrophage inflammatory protein-2 and neutrophil influx levels in BAL fluid. In an ex vivo perfused human lung, intravenous treatment of MSC MVs dramatically enhanced the rate of alveolar rate clearance, decreased lung protein porosity, and numerically reduced the number of CFU bacteria and inflammation in the wounded alveolus following severe *E.coli* pneumonia [95]. Inflammatory cytokines, invading leukocytes and the degree of pulmonary congestion, were all reduced due to prophylactic therapy with MSC EVs in rats suffering from traumatic lung injury [96].

**Fig. 3:**
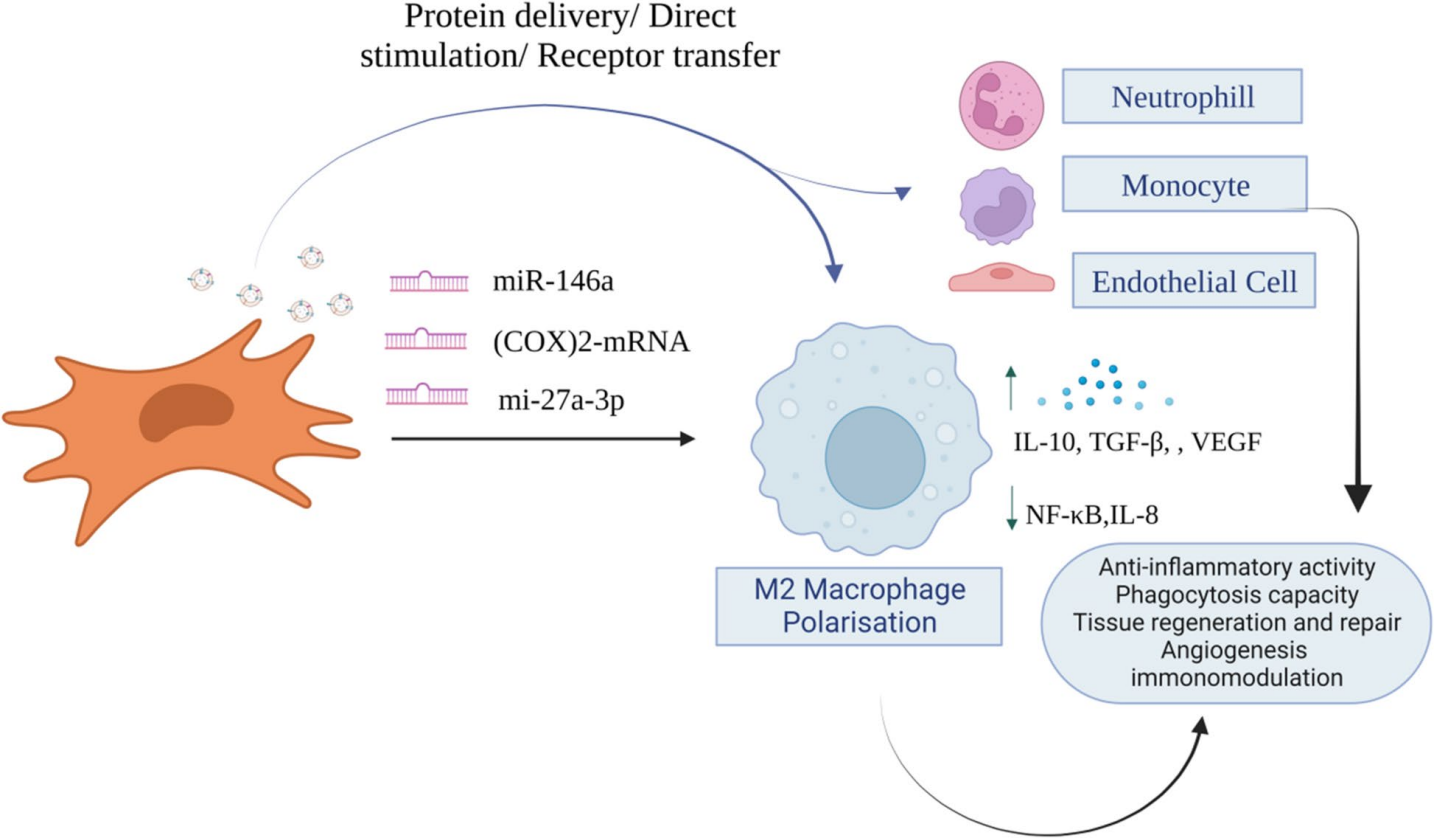
MSC-EVs can produce different kinds of miRNAs causing M2 macrophage polarisation that in turn results in increased cytokine IL-10, VEGF and TGF-β and decreased NF-κB and IL-8 expression. Furthermore, the EVs are internalised into endothelial cells, monocytes, neutrophils and macrophages. As a result of which there is less pro-inflammatory cytokine production, improved phagocytic activity and increased tissue regeneration

MSC-EVs miRNA, protein, mRNA and mitochondria are important in influencing immune responses and healing ALI lung damage. MSC-EVs have been shown to improve ALI by transferring miR-27a-3p to alveolar macrophages, which inhibits NF-κB transcription and induces M2 activation [97]. MSCs primed with IL-1β produce EVs expressing miR-146a, which generates an M2 macrophage phenotype [98]. These MSC EV-modified alveolar macrophages exhibited increased anti-inflammatory cytokine IL-10 production while decreasing inflammatory cytokines TNF-α and IL-8 release and enhanced phagocytic activity against bacteria [99, 100] (Fig. 3). MSC-EVs reduced lipopolysaccharide (LPS)-induced lung damage by upregulating angiopoietin-1 mRNA [101]. miR-27a-3p was transported from MSC-EVs to alveolar macrophages, regulating macrophage polarisation and reducing acute lung injury [97]. MSC-EVs increased the energy metabolism of acceptor macrophages and decreased silica-induced lung inflammation by transferring mitochondria from MSCs to macrophages [102]. MSC-EVs produce cyclooxygenase (COX)-2 mRNA, an enzyme that triggers the production of prostaglandin E2 [85]. Prostaglandin E2 helps switch phenotypes from pro-inflammatory M1 to anti-inflammatory M2 producing high levels of IL-10 [103, 104]. In pigs with influenza virus-induced ALI, EVs generated from bone marrow MSCs displayed anti-inflammatory and anti-influenza properties. EVs generated from porcine BM-MSCs displayed mesenchymal markers, reduced influenza virus multiplication in vitro and in vivo, and mitigated influenza virus-induced ALI [105]. MSC-EVs communicate with immune cells, causing the synthesis of TGF-β and T-regulatory cells (Tregs) [106]. In influenza virus-infected mice, Tregs increase viral elimination and healing [107, 108].

### COVID-19

**Fig. 4:**
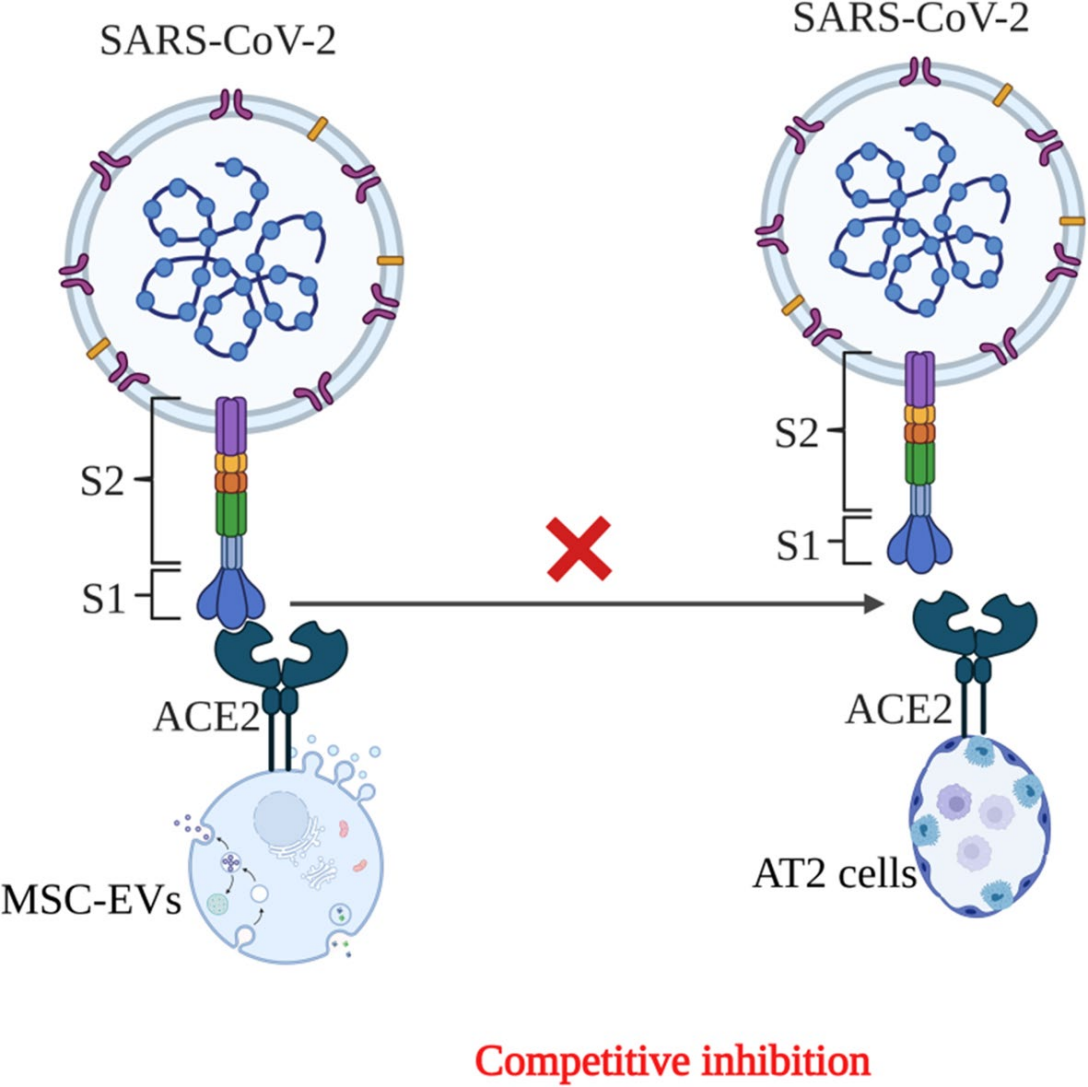
MSC-EVs expressing ACE2 receptor competitively binds to SARS-CoV-2 and prevents the binding of SARS-CoV-2 to AT2 cells

The lining of the lung alveoli is composed of a single layer of alveolar type 1 (AT1) and type 2 (AT2) cells. On lung surfaces, ACE2 receptors have been discovered, predominantly on AT2 cells and resident alveolar macrophages [109] SARS-CoV-2 binds with ACE2 receptors expressed on AT2 cells, which are the target cells of SARS-CoV-2. Alveolar cells also express the transmembrane serine protease 2 (TMPRSS2) enzyme, which is important in priming the S (spike) protein of SARS-CoV-2, which in turn facilitates infection of alveolar cells. This increases the production of pro-inflammatory cytokines and chemokines, attracting an increasing number of inflammatory macrophages and circulating immune cells into the sick alveoli, resulting in a cytokine storm (a systemic over-inflammatory condition) [110]. Moreover, cytokine storms influence AT1 and AT2 cells, lowering surfactant production; this increases the alveolar surface tension and collapse and lowers gaseous exchange, ultimately leading to ARDS [111]. ACE2 SARS-CoV-2 receptors generated on MSC-derived EVs decrease the viral infection by competitively inhibiting SARS-CoV-2 binding to alveolar cells, mainly AT2 cells [112] (Fig. 4). A study conducted by Sengupta et al. revealed that inflammatory markers and absolute neutrophilic levels were dramatically decreased. However, total lymphocyte and CD8+ counts significantly increased after 5 days of EV injection in 24 COVID-19 severe pneumonia patients treated with azithromycin and hydroxychloroquine [113].

A study by Akbari et al. demonstrated that intravenous injection of MSCs and MSC-EVs showed enormous potential in treatment for COVID-19 patients, with no adverse events during the treatment and follow-up period [114]. MSC-derived EVs exert their therapeutic function in COVID-19 by delivering protective and anti-inflammatory RNAs and proteins to damaged or activated cells in lung tissues. MSC-EVs carry a cargo which consists of different types of microRNAs. It has been reported that miR-124-3p has been involved in suppressing oxidative stress and inflammatory cytokines by binding to its receptor P2X ligand-gated ion channel 7 (P2X7). Another miR-21-5p has been associated with reducing lung cell apoptosis through inhibition of PTEN, and PDCD4-like wise miR-146a participates in transforming macrophages from pro-inflammatory to anti-inflammatory states by suppressing the NF-κB signalling pathway [115]. Hao et al. reported that miR-145 from MSC-EVs increases the phagocytic property of macrophages to enhance the clearance of pathogens at the site of infection [116]. Additionally, MSC-derived EVs increase mitochondrial activity to boost energy production in alveolar cells and increase their repairing capability in the injured lung. A study by Pacienza et al. reported that MSC-EVs reduce lung inflammation by lowering the recruitment of neutrophils and preventing macrophage polarisation via down-regulating macrophage inflammatory protein-2 levels [117].

A non-randomised clinical trial carried out in 2020 evaluated the safety and efficacy of MSC exosomes as treatment for COVID-19. Patients received a single dose of exosomes (ExoFlo™) intravenously. ExoFlo™ showed safety and efficacy with a survival rate of 83%. The ExoFlo™ treatment showed safety, help to restore oxygenation, downregulate cytokine storm and reconstitute immunity, and can be a promising therapy for treatment for COVID-19. Another double-blind, randomised controlled trial (RCT) clinical trial on “Efficacy and Safety of EXOSOME-MSC Therapy to Reduce Hyper-inflammation In Moderate COVID-19 Patients (EXOMSC-COV19)” is currently going on. The EXOSOME-MSC is being tested as adjuvant, as a complementary treatment to standard COVID-19 drugs. It will be injected to participants intravenously twice, in day 1 and day 7 of 14 days of study participation.

### Urinary tract infections

The range of infection-related renal disorders is extensive. Acute kidney damage (AKI), glomerulonephritis, tubulointerstitial nephritis, pyelonephritis, and hydronephrosis are all caused by infections through diverse pathways. AKI is among the most common manifestations, which can arise either spontaneously or in the context of earlier chronic kidney disease (CKD). AKI is a rapidly deteriorating renal functioning characterised by elevated blood urea nitrogen and plasma creatinine levels and/or decreased urinary output over hours to days [118, 119]. Most of the patients recovering from AKI have permanent renal impairment and can develop CKD [120]. MSC-EVs stimulate tissue repair by preventing pathophysiological stresses in CKD by addressing fibrosis, lowering tubular atrophy, and swelling and boosting the formation of blood vessels [121, 122]. Furthermore, it has been demonstrated that IGF-1 and IGF-1 receptor mRNA is directly secreted by MSC-EVs into renal tubular epithelial cells (TECs) along with IGF-1 receptors to stimulate kidney healing in AKI [123]. This is thought to happen due to IGF-1-induced stimulation of the Akt signalling pathway [124]. Inhibiting Sema3A significantly activated the Akt and ERK pathways, resulting in cell proliferation and resistance to AKI [125]. MSC-EVs carry other growth factors besides IGF-1 to protect the milieu of affected cells. FGF-2 has an antifibrotic impact, and knocking down FGF-2 stops tissue regeneration and promotes a fibrotic reaction [126]. ADMSC-EVs were reported to have antifibrotic characteristics resulting in reduced renal fibrosis [127, 128].

### Wound infections

Wound healing has a complicated mechanism consisting of overlapping processes like haemostasis, inflammation, proliferation, and remodelling [129]. This necessitates intercellular interaction between resident and immune cells through extracellular matrix [130]. EV from MSCs has been found to possess cytokines, chemokines, chemokine receptors, and other immune cell chemoattractant molecules. As a result, the EV may stimulate a more robust defence against infections in injured tissues [131, 132]. Human umbilical vein endothelial cells (HUVEC) were treated with ADMSC-EVs in vitro. Endothelial cells swallowed the EVs, enhancing proliferation, migration, and angiogenesis [133]. These processes were also enhanced by human umbilical cord MSC.

**Fig. 5:**
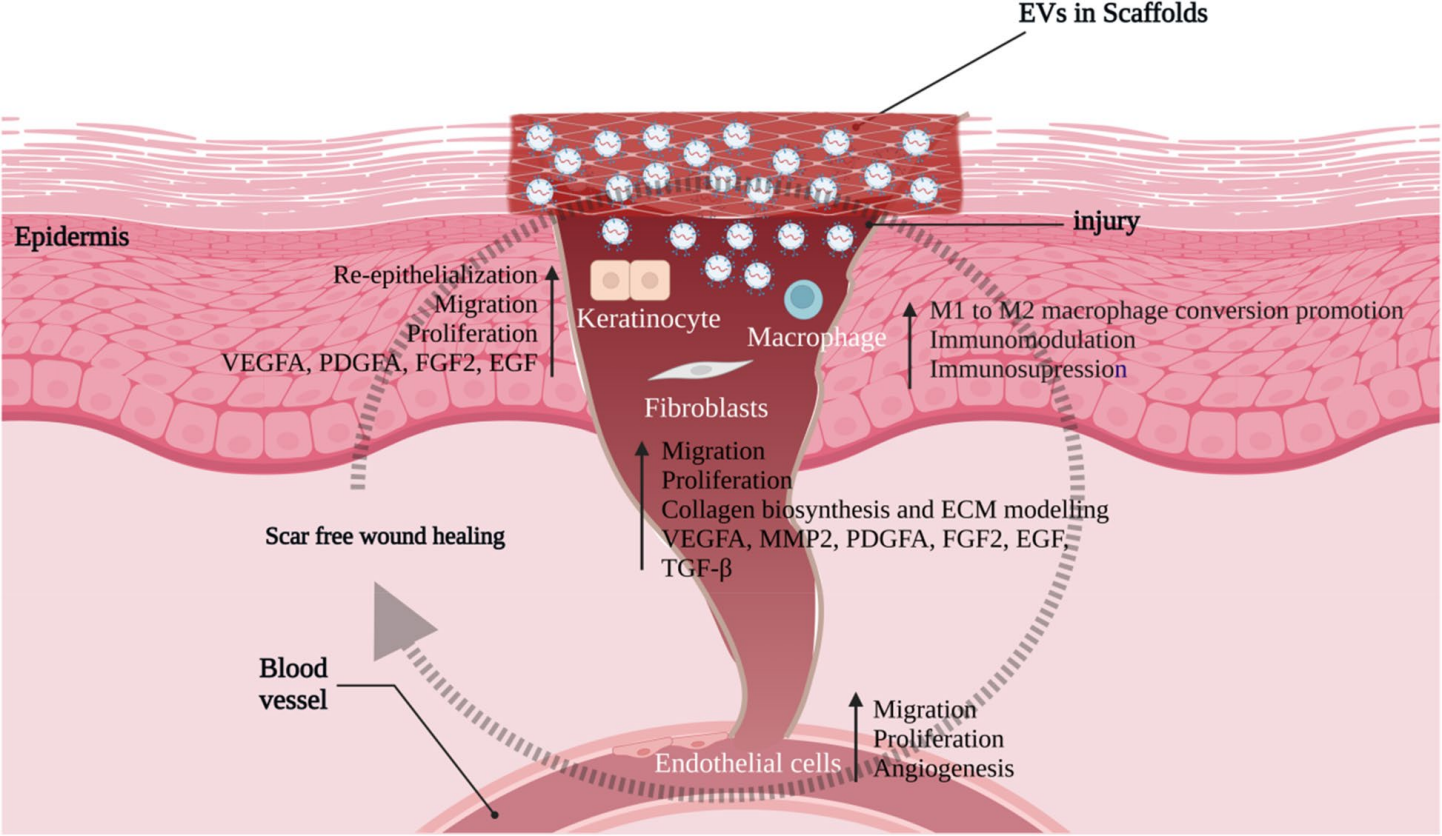
Wound healing is enhanced by the local application of scaffolds carrying MSC-EVs. These MSC-EVs enhance the effects of different cell types that are involved in wound healing. These cells include fibroblasts, keratinocytes, endothelial cells and immune cells

Furthermore, they enhanced angiogenic capacity in a rodent model of second-degree skin burns [134]. MSC-EVs can reduce the deposition of fibroblast collagen, transdifferentiation of fibroblasts to myofibroblasts, and excessive development of scars [135]. MSC-derived EVs assist the healing of diabetic foot ulcers (DFUs) by delivering bioactive molecules. In addition, as a vehicle for non-bioactive compounds such as antibiotics, it can limit bacterial activity and speed up wound healing in bacteria-associated DFUs [136]. DFUs and wounds can be treated effectively with optimised MSC-derived exosomes. Exosomes from MSCs pretreated with salidroside exhibited regeneration of diabetic wounds [137]. According to prior research, ADMSC exosomes integrated with a hydrogel exhibit healing, antimicrobial, and exosome releasing properties [138] (Fig. 5). Exosomes produced from TSG-6-enhanced MSCs inhibited the development of scars by lowering inflammation and preventing collagen deposition [139].

### Intestinal infections

Intestinal tract homeostasis is crucial owing to the direct exposure to the digestive residue, foreign antigens and millions of pathogens. The intestinal mucosal barrier plays a pivotal role in detecting, clearing the microbial debris, and maintaining a peaceful coexistence. The intestinal defence system consists of the intestinal epithelial cells (IECs), mucus layer and other innate immune system cells.

EVs participate in vascular and epithelial barrier function in wound healing and inflamed intestines. Inflammatory bowel disease (IBD) is a group of persistent, emerging inflammatory diseases of the intestine that are marked by recurring abdominal discomfort, prolonged diarrhoea, and bloody faeces with mucus and pus. There has been a sharp increase in the abundance of Bacteroidetes and Proteobacteria, primarily *E.coli* in IBD [140]. The severity of colitis was reduced after intravenous administration of bone marrow MSC-EVs, as demonstrated by a reduction in the disease activity index (DAI) and colon damage. The study provided evidence for BMSC-EV’s prospective therapeutic benefits in TNBS-induced colitis, such as inflammatory regulation, oxidative stress suppression, and apoptosis abatement [141]. IBD affects around 3.5 million people worldwide, with its aetiology and pathogenesis still unclear [142]. Several studies of IBD patients report dysbiosis of the gut microbiota with abnormal metabolism of IECs due to a fluctuating abundance of cargo in EVs and MVs [143]. In an active IBD intestinal microenvironment, an increase in the recruitment of innate immune cells such as dendritic cells, neutrophils, monocytes, macrophages, and T cells occurs. As a result, EVs are tightly associated with macrophages in IBD. Additionally, EVs have been reported to have immunosuppressive effects, such as suppress the DCs activation to induce immune tolerance and contributing in Treg activation [144], which will eventually secrete exosomes targeting pro-apoptotic caspase-12 alleviating IBD in mice [145]. In IBD, IECs secrete epithelial cell adhesion molecule-dependent EVs with high TGF-β1 levels, thus maintaining the intestinal tract immune balance and decreasing the IBD severity [146].

**Fig. 6:**
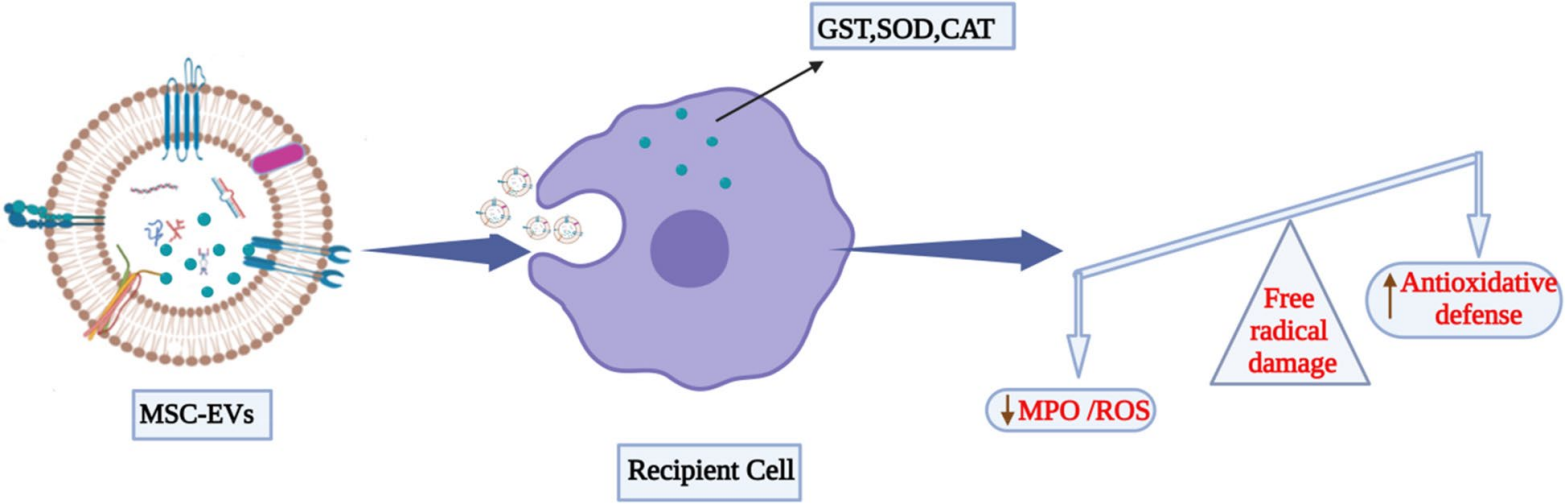
MSC-EVs have the ability to deliver antioxidant enzymes like superoxide dismutase (SOD), glutathione S-transferase (GST) and catalase (CAT) directly into the target cells. These anti-oxidant enzymes effectively reduce oxidative stress and inflammation in intestines

Administration of EVs derived from umbilical cord-MSCs has alleviated colitis in mice [147]. In a recent study, human adipose mesenchymal stem cell-derived exosomes (hADSC-Exo) stimulate the proliferation and regeneration of Lgr5-ISCs and epithelial cells and ameliorate TNF-α induced inflammatory damaged mice colon organoids. hADSC-Exos are a potential treatment for IBD as they protect intestine integrity and activate the intestine epithelial cells [148]. Another study reported the MSC-EV-based alleviation of colitis by inducing suppression of colon macrophages. Treatment with MSC-EVs significantly reduced activation of IL-7 and iNOS-signalling pathways in colon macrophages, ultimately resulting in attenuated production of IL-1β, IL-6, TNF-α, and increased secretion of IL-10, leading to colitis alleviation [147]. To examine the effects and possible mechanism of EVs derived from bone marrow MSCs in ulcerative colitis (UC) treatment, an in vitro model of LPS-treated macrophages, and an in vivo dextran sulphate sodium (DSS)-induced mouse model were established to impersonate UC. EVs promote proliferation and dampen the inflammatory response in LPS-induced macrophages. In the in vivo model, administered EV ameliorated the UC symptoms by ameliorating colon mucosa damage and severity, disease activity index, and weight loss while increasing colon length. EVs from bone marrow MSCs attenuated ulcerative colitis by endorsing M2 macrophage polarisation [149]. EVs derived from human placental MSCs have also been utilised to treat colitis, which markedly reduced intestinal inflammation and oxidative stress by dampening the activity of myeloperoxidase (MPO) and reactive oxygen species (ROS) [150] (Fig. 6).

### Sepsis

Sepsis is a severe organ dysfunction due to a deregulated host response to infection. Presently, sepsis incidence is approximately 270 cases per 100,000 persons per year, with an approximate 26% mortality rate [151]. Sepsis usually involves an imbalance of the anti- and pro-inflammatory components of the immune system, characterised by immune system hyperactivation followed by an aggravated anti-inflammatory state, ultimately leading to immunosuppression [152]. It is accompanied by coagulation and haemodynamic alterations and cellular injuries leading to the development of multiple organ dysfunction (MOD). EVs are released from cells upon activation and apoptosis and will express membrane epitopes which are specific to their parental cells. In sepsis models of mice and patients, high numbers of EVs in the blood are reported compared to healthy individuals by flow cytometry and NTA techniques [153]. All immune cells, endothelial cells, RBCs, and platelets are reported as sources of EVs in the blood in both disease and healthy states. Septic EVs contribute to the spread of inflammation and exert pro- and anti-inflammatory characteristics. A massive cytokine storm characterises early-phase sepsis in the circulation wherein EVs are identified as carriers of chemokines, cytokines, and growth factors [154, 155]. During systemic inflammatory conditions, released EVs contain DAMPs, including heat shock proteins (HSPs), histones, and high-motility group box-1 (HMGB1) [156, 157]. C-reactive protein (CRP), an acute phase protein, is another pro-inflammatory protein in septic EVs. Hepatocytes primarily secrete CRP in response to systemic inflammatory conditions and tissue damage. Septic EVs have also been reported to contain differentially expressed miRNAs associated with inflammatory pathways. EVs can influence clinical outcomes as well as predict survival. However, beneficial and detrimental roles are associated with sepsis depending on the cellular source and the disease phase during which the EVs are being studied.

In animal models, MSC-EVs have been proven to improve the outcome of sepsis. miRNAs from MSC-EVs vigorously exert their role in sepsis. miR-21 in MSC-EV was upregulated in IL-1β-stimulated MSCs inducing M2 polarisation of macrophages in vitro and in vivo sepsis [158]. miRNA-146a was also upregulated in MSC-EVs primed with IL-1β, which effectively induces M2 polarisation by altering TRAF6, IRF5 and IRAK1 signalling [98]. These studies support the notion of pretreating MSCs with pro-inflammatory cytokines, eventually augmenting the immunomodulatory function of MSCs.

MSC-Exo therapy may complement as adjuvant therapy in sepsis-induced acute kidney, liver, and cardiovascular system injuries. The capability of MSC-Exos has been validated to rescue the renal function in sepsis [159]. Kidney morphology was found to be more intact after MSC-Exo intervention in caecal ligation and puncture (CLP)-induced sepsis mice. A reduced inflammatory cell infiltration, decreased kidney interstitial oedema, and higher integrity of brush borders were observed in HE staining kidney tissues [160]. Renal function was also restored, which was confirmed by a blood test. MSC-Exos have been reported to show hepatic protection in acute liver injury demonstrated by improved hepatic function indicators, a lower degree of hepatocellular necrosis and inflammation [161]. The therapeutic effects of MSC-Exos lie in innate immune system modulation. MSC-Exos participate in hepatocyte haemostasis maintenance by inhibiting cell apoptosis. BMSC-Exos reportedly reduce apoptosis of hepatocytes by increasing levels of autophagy marker proteins, an increasing number of autophagosomes and microtubule-associated protein 1A/1B-light chain 3, Beclin-1 [162]. MSC-Exos are reported to suppress inflammation and maintain calcium homeostasis under septic conditions. Wang et al. (2015) injected MSC-Exos intravenously in septic mice. It resulted in overall mice survival by inhibiting cardiomyocytes’ death and attenuating excess inflammation via miR-233. MSC-Exos represent an efficacious cell-free therapeutic modality for sepsis treatment depending on its anti-inflammatory and anti-apoptotic activities [163].

### Pharmacokinetics of EVs

It is essential to understand the pharmacokinetics of EVs in order to deliver therapeutic molecules to precise sites in the body. The duration during which large concentrations of EVs are kept in the bloodstream after intravenous injection is significant. EVs generally concentrate in the organs of reticuloendothelial system (RES), also called as mononuclear phagocyte system (MPS) [164]. The best way to enhance EV pharmacokinetics is to prevent MPS accumulation. The MPS captures nearly all of the EVs when they are systemically delivered. The quick removal of EVs from the bloodstream is caused by MPS macrophages. Additionally, since macrophages have phosphatidyl serine (PS) binding receptor molecules, the negatively charged surface of EVs, which is derived from the similarly negatively charged PS, is what drives the majority of macrophage uptake [165, 166].

The pharmacokinetics of EVs is also greatly influenced by their source. The reason behind this is that every EV has a unique makeup. EVs from various cell types demonstrated somewhat varied biodistribution. The pharmacokinetics is greatly impacted by the mode of administration. Intravenous injection of EVs causes fast removal from blood and build-up in MPS-related tissues, as was previously stated. The novel method for delivering drugs into the brain is through intranasal administration. Oral administration, which can use EVs obtained from food, is a promising approach as it is minimally intrusive, yet it is still unknown if EVs can enter the bloodstream by intestinal absorption while retaining their integrity [167, 168].

Another useful strategy for avoiding MPS capture may be PEGylation. PEG is a hydrophilic polymer that, when coupled to nanoparticles, forms chains that cover the exterior of these nanoparticles. The contact between nanoparticles and proteins or cells can be diminished, and the half-life can be extended owing to the steric hindrance influence of PEG chains. Such methods can be used to enhance the pharmacokinetics of EVs [164]. Matsumoto et al. revealed that injecting a liposome containing PS or phosphatidylglycerol prior to systemic administration increased EVs in blood circulation [166]. These methods are useful for enhancing the pharmacokinetics of EVs and enabling tailored distribution to particular cells or organs.

### Toxicity and tumorigenicity tests

MSC-EVs have already undergone numerous preclinical and clinical trials. MSCEV tolerance has been documented in a large number of studies; however, security-related concerns have received far less attention. However, a study was conducted on the toxicity of MSC-EVs. Hyun et al. performed toxicity assays like in vitro photosensitisation, local lymph node assay, acute oral toxicity, and eye and skin irritation. Their results suggested that the MSCEVs are risk-free and have no side effects. The ability of MSC-EVs to shield cells from UV irradiation damage was demonstrated [169]. EVs produced from wild-type and modified HEK293T cells (non-MSC-EVs) did not cause any toxicity or a significant immunological reaction in immune-competent C57BL/6 mice [170]. To examine the safety of hucMSC exosomes in vivo, rats with acute myocardial infarction were injected intravenously with the exosomes. In addition to protecting against weight reduction, hucMSC exosomes had no negative impacts on liver or kidney function [171]. In vitro toxicological evaluations were used to determine the safety of EVs derived from MSCs or bovine milk. Neither the alkaline comet assay nor the micronucleus assay revealed any genotoxic effects from either MSC-EVs or bovine milk-derived EVs [172].

Exosomes from colorectal cancer stem cells increased the lifespan of bone marrow-derived neutrophils and caused them to have a protumoural character. Interleukin-1 expression was increased by tumour exosomal triphosphate RNAs via a pattern recognition-NF-κB signalling axis to maintain survival of neutrophils [173]. Exosomes derived from melanoma that carry oncogenic molecular reprogramming cause naive MSCs to turn into melanoma-like cells that overexpress programmed cell death protein 1(mMSCPD-1+). In vivo, oncogenic factors are expressed, and tumour growth is induced by exosomes and mMSCPD-1+ cells [174]. A study by Gu et al. revealed that MSC-EVs enhanced the proliferation and metastatic properties of gastric cancer cells ex vivo. The epithelial-mesenchymal transition was reported to be induced by MSC-EVs, which improved the migration and proliferation of HGC 27 cells. The tumorigenicity of gastric cancer cells was also increased by MSC-EVs [175].

## Conclusion

MSC-EVs have a potential role in therapeutic regimens for infectious diseases, including intestinal infections, respiratory infections, and sepsis in recent years. The therapeutic mechanisms include immunomodulation, direct antimicrobial effects, and tissue repair. MSC-EVs reportedly exert their effect through the transfer of mRNAs, proteins, and miRNAs. EVs secreted by MSCs have emerged as a cell-free alternative to MSC-based therapies owing to their low risk of immunogenicity, tumorigenicity, and higher safety. Even though EV-based therapies are more attractive in terms of safety, specific concerns should be addressed for their clinical use. There is a wide variation and a lack of standardisation procedures regarding MSC expansion or EV purification methods. Protocol standardisation and potency tests are crucial for clinical grade exosomes or EV preparations. However, further investigations are required to fully elucidate the immunomodulation mechanism and the EV cargo responsible for the cellular level alterations specific to clinical conditions. Furthermore, toxicity profiling, which may include evaluations of immunological responses, need to be carried out on all EVs preparations.

## Data Availability

Not applicable

## Abbreviations

EVs: Extracellular vesicles
MSCs: Mesenchymal stem cells
MSC-EVs: Mesenchymal stem cell-derived extracellular vesicles
mRNA: Messenger RNA
miRNA: Micro RNA
MVBs: Multivesicular bodies
S1P: Sphingosine-1-phosphate
ESCRT: Endosomal sorting complex required for transport
Alix: ALG-2-interacting *protein* X
HRS: Hepatocyte growth factor-regulated tyrosine kinase substrate
TSG-101: Tumor susceptibility gene 101
HIV: Human immunodeficiency virus
MVs: Microvesicles
MMPs: Matrix metalloproteinases
ISEV: International Society for Extracellular Vesicles
sEVs: Small EV
m/lEVs: Medium/large EVs
TEM: Transmission electron microscope
NTA: Nanoparticle tracking analysis
AFM-IR: Atomic force microscope-infrared spectroscopy
ARF6: ADP-ribosylation factor 6
ERK: Extracellular signal regulated kinase
MLCK: Myosin light chain kinase
VPS4: Vacuolar protein sorting-associated protein
ROCK: Rho-associated coiled-coil containing kinases
ILVs: Intraluminal *vesicles*
*CHMP4*: Charged multivesicular body protein
TIM4: T-cell immunoglobulin mucin 4
EGF: Epithelial growth factor
TSP: Thrombospondin
MHC: *Major histocompatibility complex*
BALF: Bronchial-alveolar lavage fluid
ALI: Acute lung injury
ARDS: Acute respiratory distress syndrome
BPD: Bronchopulmonary dysplasia
IT: Intratracheally
LPS: Lipopolysaccharide
COX: Cyclooxygenase
TGF-β: Transforming growth factor
Tregs: T-regulatory cells
AT1: Alveolar type 1 cell
AT2: Alveolar type 2 cell
ACE2 receptor: Angiotensin-converting enzyme 2 receptor
TMPRSS2: Transmembrane serine protease 2
P2X7: P2X ligand-gated ion channel 7
AKI: Acute kidney injury
CKD: Chronic kidney disease
IGF: Insulin-like growth factor
TECs: Tubular epithelial cells
ADMSCs: Adipose-derived mesenchymal stem cells
HUVEC: Human umbilical vein endothelial cells
DFUs: Diabetic foot ulcers
IECs: Intestinal epithelial cells
GI: Gastrointestinal
MOD: Multiple organ dysfunction
DAMPs: Damage-associated molecular patterns
HSPs: Heat shock proteins
HMGB1: Histones and high-motility group box-1
CRP: C-reactive protein
TRAF6: Tumour necrosis factor receptor (TNFR)-associated factor 6
IRF: Interferon regulatory factor
IRAK1: Interleukin-1 receptor-associated kinase 1
TLR: Toll-like receptors
HAP: Hemagglutinin protease
IBD: Inflammatory bowel disease
DCs: Dendritic cells
hADSC-Exo: Human adipose mesenchymal stem cell-derived exosomes
UC: Ulcerative colitis
DSS: Dextran sulphate sodium
MPO: Myeloperoxidase
TNBS: 2,4,6-Trinitrobenzene sulfonic acid
ROS: Reactive oxygen species
SOD: Superoxide dismutase
CAT: Catalase
GST: Glutathione-s-transferase
CLP: Cecal ligation and puncture
RES: Reticuloendothelial system
MPS: Mononuclear phagocyte system
